# Mechanistic Impact of Zinc Deficiency in Human Development

**DOI:** 10.3389/fnut.2022.717064

**Published:** 2022-03-09

**Authors:** Azhar Hussain, Wenting Jiang, Xiukang Wang, Shumaila Shahid, Noreena Saba, Maqshoof Ahmad, Abubakar Dar, Syed Usama Masood, Muhammad Imran, Adnan Mustafa

**Affiliations:** ^1^Department of Soil Science, The Islamia Diversity of Bahawalpur, Bahawalpur, Pakistan; ^2^College of Life Sciences, Yan'an University, Yan'an, China; ^3^Qaid-e-Azam Medical College, Bahawal Victoria Hospital, Bahawalpur, Pakistan; ^4^Clinical Fellow Pediatric Nephrology, Children Hospital and Institute of Child Health Multan, Multan, Pakistan; ^5^Soil and Water Testing Laboratory, Khanewal, Pakistan; ^6^Faculty of Chemistry, Institute of Chemistry and Technology of Environmental Protection, Brno University of Technology, Brno, Czechia; ^7^Department of Agrochemistry, Soil Science, Microbiology and Plant Nutrition (FA), Mendel University, Brno, Czechia; ^8^Institute of Environmental Studies, Charles University Prague, Prague, Czechia

**Keywords:** Zn deficiency, detoxification, apoptosis, deoxyribonucleic acid, reactive oxygen species

## Abstract

Zinc (Zn) deficiency in humans is an emerging global health issue affecting approximately two billion people across the globe. The situation prevails due to the intake of Zn deficient grains and vegetables worldwide. Clinical identification of Zn deficiency in humans remains problematic because the symptoms do not appear until impair the vital organs, such as the gastrointestinal track, central nervous system, immune system, skeletal, and nervous system. Lower Zn body levels are also responsible for multiple physiological disorders, such as apoptosis, organs destruction, DNA injuries, and oxidative damage to the cellular components through reactive oxygen species (ROS). The oxidative damage causes chronic inflammation lead toward several chronic diseases, such as heart diseases, cancers, alcohol-related malady, muscular contraction, and neuro-pathogenesis. The present review focused on the physiological and growth-related changes in humans under Zn deficient conditions, mechanisms adopted by the human body under Zn deficiency for the proper functioning of the body systems, and the importance of nutritional and nutraceutical approaches to overcome Zn deficiency in humans and concluded that the biofortified food is the best source of Zn as compared to the chemical supplementation to avoid their negative impacts on human.

## Introduction

Nutritional deficiency is an emerging global crisis in the developing and the developed world which cause deficiencies in growth, immune functions, cognitive and motor progress, behavior, and intellectual performances in humans specially children. Zinc is a nutritional element because of its prime role in the human body, such as binding material in biological process (1). Zinc performs structural, catalytic, and regulatory functions in the human body through an array of enzymes for proper functioning (2). Zinc acts as a component of several metalloenzymes in living organisms, hemoprotein enzymes, and nuclear enzymes (3).

### Global Prevalence and Significance of Zn Deficiency in Human

In 1960, the importance of Zn for animal growth focusing on multiple species was studied and documented. However, the past decades have addressed the Zn research in areas to use Zn supplements to overcome the health issues in humans. However, the first experiment from the Middle East indicated its importance in disease development in humans. Zn deficiency can cause disease in humans that leads toward important clinical issues (4). Moreover, the deficiency of zinc is prevalent in the old age population because of lower Zn in their nutrition, change in lifestyle and 65% of the world's population is facing the food starvation overall and lives under destitution line (5).

### Zinc Deficiency and Clinical Manifestations in Human

Micro-nutrient deficiencies, such as Zn, greatly contribute to a number of diseases and lead toward adverse functional disorders, such as blindness, cognitive losses, decreased IQ level, stunting, premature mortalities during pregnancy, and increased exposure to infectious diseases during pregnancy. There are numerous reasons for the Zn deficiency mainly insufficient Zn absorption by the human body and consumption of low Zn meals, presence of Zn inhibitors, and excessive Zn loss during diarrhea (6). Globally, it is estimated that two billion individuals are in danger of clinical illness due to Zn deficiency. Current estimates have revealed that about 17.3% of the World's population is at risk by using Zn deficient food commodities (7). Life-threating medical disorders, such as cancer, diabetes, and chronic and oxidative stresses, are more prominent in adults in response to Zn deficiency (8). Short-term memory, structural malformation of brain, reduction in thinking activity, and behavioral problems in adults also demonstrate the insufficient Zn concentrations in human food (9). Severe Zn deficiency causes hyperzincuria, increased hemolysis leading to the reduction in growth, deficient immunity system, infertility, hyperammonemia, hypogonadism in men, and thymic atrophy (10).

In humans, the most common type of inherited Zn deficiency is Acrodermatitis enteropathica (AE), which is an autosomal recessive disorder (11). Genes responsible for Zn homeostasis may lead to AE in the absence of Zn and may cause mutations in gene. Other reasons for AE include impaired or insufficient absorption of Zn from the intestine leading toward alopecia, dermatitis, and gastrointestinal track misfunctioning in Zn uptake. Moreover, disturbances in neuropsychological system, such as depression, behavioral problems, and disturbance in immune system, are common symptoms in AE due to intestinal transporter's mutation and becoming the most serious type of the inheritably acquired disorder in humans (12).

### Concentration Levels of Zinc in Human Body

The young human body contains 2–3 g/kg of Zn of body weight above 60 kg. The total amount of Zn in all body fluids and tissues has been recorded to almost 60%, in skeletal muscles, ~30%, and blood plasma contains 0.l% Zn (13). Its supply to body from the ingested food depends upon the concentration of Zn in food and its bioavailability in body through the digestion process. It has been studied that mixed western diet has the ability to provide about 20–30% of total contained Zn to body for the functioning (14). There are several agents that have the ability to reduce Zn absorption in the body, such as phytates, copper, iron, and calcium are named as Zn absorption inhibitors (15). The recommended daily intake of Zn from diet varies between 10 and 15 mg in adults but an extra 5 and 10 mg amount is required by the pregnant adults and lactating mothers (16). Generally, there are some chemical approaches to overcome the lower Zn levels in humans as Nutraceuticals or chemicals supplementation, which are given in the form of chemical compounds of specific nature as capsules, syrups, and pills. The chemical approaches are equally adopted by the developed and developing world providing an instant supplement of the Zn. The shifting of mechanized agriculture toward organic agriculture in the developed world has strengthened the idea of biofortified food, especially for the micro-nutrients. The major benefit of the biofortified food is that it provides higher concentrations of the micronutrients as compared to non-biofortified food. The present review emphasizes the importance of Zn in relation to human physiology, body functioning, Zn deficiency-induced diseases, immune system response in Zn fortification, and the approaches to overcome Zn deficiency.

## Zinc and Human Physiology

### Physiological and Biological Role of Zinc

Zinc plays significantly important physiological and biological roles in human body. It is estimated that more than 300 enzymes of human body need Zn for physiological and biological activation directly or indirectly. The important biological processes, such as catalytic, structural, and regulatory functions of the body, require an adequate amount of Zn for proper functioning (8). Zn also performs another important role as a stabilizer of the tertiary structure for more than 300 molecular proteins, such as Zn finger proteins, and regulates various transcription factors in human body. These finger proteins control the gene expression of a variety of growth factors, immune response mediators, and steroid receptors through binding to proteins, RNA, and DNA (8). Zn ions also control the synthesis and functioning of peptide hormones, expression of genetic information, and physiological maintenance of chromatin and bio-membranes (16).

### Role of Zinc in the Endocrine System

Zinc as a growth mediator in human body is important for bone metabolism due to the presence of its higher concentrations in the bone along with acting as an important co-factor in hormonal synthesis through the endocrine system. Moreover, it is responsible for the binding of the produced hormones to their specific receptors by enhancing receptors in numbers (17). The endocrine system comprises of different active glands, such as thyroid and parathyroid, testes, the ovaries, and pituitary glands (17). Zinc deficiency can cause oxidative stress, which leads to dysfunction of the thyroid glands in humans consuming Zn deficient diet or have higher Zn excretions through urine. As the Zn level significantly reduced due to hypothyroidism (a thyroid disease), the Zip 10 (Zn transporting proteins) showed a positive correlation with renal and intestinal thyroid hormone (18). Another important hormone Insulin (responsible for carbohydrates metabolism in body) is stored in crystalline form with Zn in the pancreas. Zn deficiency not only impairs the structure of the insulin but also reduces the efficiency and activity of insulin. It also affects the insulin pathway in a number of ways by phosphorylating the beta subunit of insulin receptor and glycogen synthesis kinase inhibition (19).

Zinc is important for improving oxytocin stability by improving divalent metal interaction with oxytocin. It also modifies the binding sites for cellular receptor required by oxytocin (20). Thyroid hormone conversion is stimulated by Zn, such as thyroxine into tri-iodothyronine and its deficiency causes hypo-thyroids, which reduced the thyroid hormone production. Thymulin is a non-peptide hormone released by thymic epithelial cells that need Zn for biological reactions (21). Zn also regulates the signal transduction, which binds as a second messenger with a specific type of protein (22).

### Role of Zinc in the Immune System

Besides its important role in improving growth and physiological functions, Zn also regulates the body immune system through hormonal balance (23). Zn is a key component of thymic hormone functioning that is responsible for facilitating and controlling lymphocytes maturation for various neuronal functions (24) and aids immune system development through cell production, DNA replication, and cell division. The mechanisms responsible for immune system response include adaptive and innate immunity (25). The immune system of an individual under Zn deficient conditions enhances cytotoxic cytokine (ROS generators), impair hematopoiesis (blood cellular component formation), humoral immunity (secreted antibodies), survival and function of the immune cell may lead toward cancer (26).

Furthermore, Zn deficiency adversely affects the body defense mechanisms, i.e., activation of macrophages, natural killer cells, polymorphonuclear cells, and complement cascade (27). Supplementation of Zn increases the adaptive and innate immunity against the infection of entero-toxigenic *Escherichia coli* (*E. coli*) due to the rise of the C3 complement system, boosts T-cell functionality and phagocytosis (28). Interestingly, immune function alterations during Zn deficiency may appear as immune senescence, such as increased cellular inflammation, thymic atrophy, and impaired humoral and cellular immune responses (29).

### Nutritional Recommendation of Zinc in Human Development

Plants, animals, and humans require Zn as an essential nutrient for their normal development and reproductive growth. Plants required Zn to attain proper fruit size, enhance crop productivity, and maximize crop yields (30). Grain crops, such as cereal and legumes, are good sources of Zn supplementation for humans. Among human nutrition, Zn plays a great role to control the effective cellular functions and improve their immunity. Deficiency of Zn can cause skin diseases, muscular system impairment, short-term memory, and hair loss. As the human body has no ability to store Zn like other nutrients, its deficiency is therefore a prime cause of infertility in humans. Hence, it is a challenge to detect and diagnose Zn deficiency in the human body by the measurement of Zn level in body cell parts (31). The average amount of Zn that is needed by adults, men, and women is estimated as 11 and 9 mg of Zn per day, respectively, as described in Table 1. Women require 13–14 mg of Zn on daily basis during pregnancy and after baby birth. New-born children of age (7 months to 3 years) require 3 mg, 4–8 years need 5 mg, and 9–13 years need 8 mg of Zn on daily basis (32). About 43% of children of age 3–5 years in South Africa (33) and in 20% of children (6 months to 12 years) in Iran (34), it was observed that the infants are more susceptible to Zn deficiency. Pre-term infants are more susceptible to Zn deficiency in the first months of their life because they have low storage of hepatic Zn and a low capacity to absorb intestinal Zn (35). The Zn ranges described in Table 1 have been provided by WHO, FAO, IOM and EFSA in different years (36–39).

**Table 1:** Different age groups with daily Zn recommendation among different age groups Zn bioavailability (mg/day).

**Population and age (year)**	**Zinc requirements daily reference intakes**			
	**(WHO 1996)**	**(WHO/FAO 2001)**	**(IOM 2006)**	**(EFSA 2014)**
**Infants**				
0–1	0.6	5.6	2–3	2.4
**Children**				
1–3	2.73	5.5	3	3.6
4–10	3.73	6.5	5	6
**Male**				
9–13	4.66	9	8	8.9
14–60	6.53	13	11	11.8
>60	6.0	9.4	11	11
**Female**				
10–12	3.96	8–9	8	8.9
12–60	5.14	10	8	9.9
>60	5.12	6.5	8	9
**Pregnant women**				
18–50	9.5–10	10–12	11–13	10–13
**Lactating women**				
19–50	10.4–11.6	9–12	12	12

*FAO, Food and Agriculture Organization; IAEA, International Atomic Energy Association; WHO, World Health Organization; IOM, Institute of Medicine; EFSA, European Food Safety Agency*.

## Dietary Sources of Bioavailable Zinc

Dietary Zn intake is of prime importance to ameliorate Zn deficiency-related disorders in humans through legumes and cereals as these are rich sources of Zn. Dietary Zn sources and respective concentrations of Zn are described in Table 2. Sustainable food processing should be practiced in order to assure health and safety and food processing should not reduce the nutritional quality of the product specifically micronutrients. Meat and polished cereals have about 40–60 mg/kg Zn amount due to high fat content and low extraction rate. The foods, such as fruits and green vegetables, have moderate amount of Zn content while alcohol has low amount of Zn availability (43). The dry and dairy products are a rich source of Zn contents (44).

**Table 2:** Zn rich foods with Zn concentration.

**Food group**	**Zn content of different foods**	
	**mg/100 g**	**mg/100 kcal**
Beef	4.2–6.1	2.7–3.8
Chicken	1.8–3.0	0.6–1.4
Seafood	0.5–5.2	0.3–1.7
Eggs	1.1–1.4	0.7–0.8
Dairy products	0.4–3.1	0.3–1.0
Dry foods	2.9–7.8	0.5–1.4
Bread	0.9	0.3
Cereals	0.5–3.2	0.4–0.9
Beans	1.0–2.0	0.9–1.2
Refined cereal	0.4–0.8	0.2–0.4
Fermented cassava root	0.7	0.2
Tubers	0.3–0.5	0.2–0.5
Vegetables	0.1–0.8	0.3–3.5
Fruits	0–0.2	0–0.6

***Source:** [Krause et al. (40); Pennington (41); Brown and Baker (42)]*.

## Zinc Deficiency-Triggered Defense Mechanisms

### Impact of Zinc on Apoptosis

Zinc is important for its essential roles in the interaction and metabolism of malignant cells and mainly in apoptosis (45). Apoptosis is a biological regulation process to control programmed cell death in many biological processes, such as involution, remodeling, development of tissue and to remove mutant, superfluous, and moderately damaged cells on exposure to toxic substances (46). Apoptosis occurs in two distinct phases, i.e., the bio-chemical signaling pathway to commit a cell suicide and the executional phase is described by morphological changes in a cell leading to death (16).

The activation of the *p38* gene, certain caspases, and proteases are an important component in apoptosis (47). The complex tertiary structure stabilization of specific DNA binding domain of *p53* gene required Zn (48), which regulates *p53* activity by *in vivo* pathway (49). Zn deficiency in the human body acts as an inhibitor for apoptosis-induced programmed cell death (50). Apoptosis is assumed as the important mechanism of cell death in case of any toxic material invades the body. The irregularities in apoptosis provide pathogen-like mechanisms in several diseases, such as autoimmune diseases, neurodegenerative disorders, cancer, and acquired immune deficiency syndrome (16).

Low levels of Zn in numerous body cells, i.e., testicular, glioma, fibroblasts, T cell precursors, and hepatocytes, can expedite apoptosis. The balance between cell death and the cell formation of an organism is controlled by numerous Zn channels to maintain optimal amount and intra-cellular movements of Zn (50). Moreover, Zn deficiency can trigger the apoptosis by disrupting cell growth factors and signal-transduction pathways intermediated by tyrosine kinase (51). Zinc deficiency inhibits the neuronal proliferation of cells, which has been investigated in human neuroblastoma cells (IMR-32) and primary cultures of differentiated cortical neurons in rats.

#### Impact of Zinc in Neuronal Death

**Figure 1:**
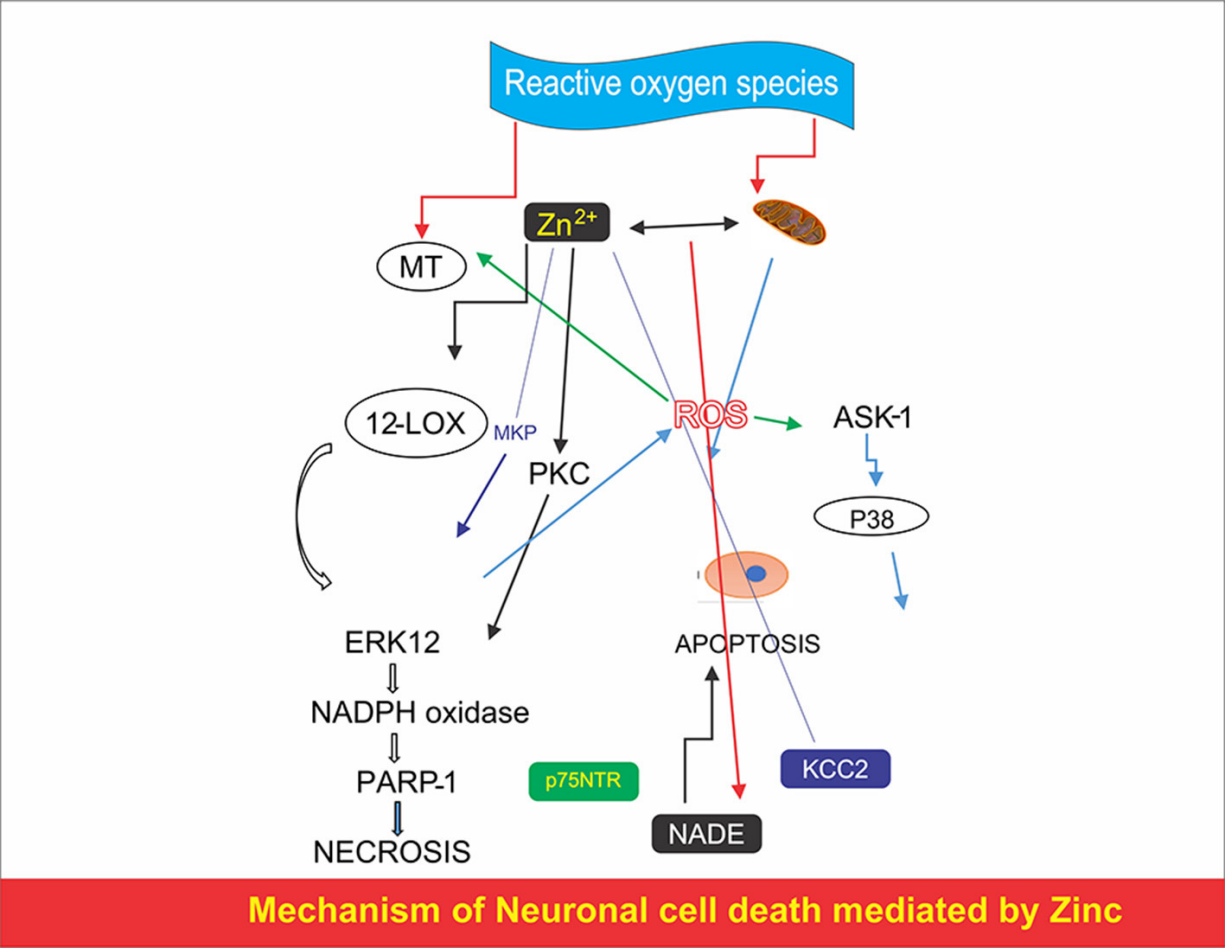
Mechanism of neuronal cell death mediated by zinc.

Acute Zn deficiency can lead toward programmed neuronal death in quiescent neurons or mature neurons. The Zn^2+^ addition to the somatic cell, possibly mediated by a maximum of Zn^2+^ translocation from presynaptic vesicles and Zn^2+^ release from animate things store, will trigger future neuro generative sign as described in Figure 1. The number of necrobiosis signs Zn^2+^ appeared in mitochondria which leads toward mitochondrial necrobiosis as function of mitochondrial impairment and aerobic pressure. Free Zn^2+^ is obsessed by mitochondria leads toward mitochondrial prospective impairment, unleashes the oxidative damage to the neurons through the production of reactive oxygen species (ROS), pro-apoptotic proteins generation, and initiation of mitochondrial growth (52). Numerous somatic cell downstream necrobiosis pathways were found to be Zn^2+^ dependent. Chemical agent or microglia-induced Zn^2+^ release has the ability to activate 12-lipoxygenase (12-LOX) leading toward *p38*-dependent improvement in K^+^ effluence *via* new put of Kv2.1 channel.

Microglia-resultant reactive chemical elemental species react ROS and activate the well-characterized apoptotic indicator regulation as kinase-1 (ASK1)/*p38*-dependent, Kv2.1-mediated K^+^ current surge (53). Additionally, Zn^2+^ has shown to be essential for *p75* neurotrophin receptor (NTR)-mediated cell death and seems to reduce K^+^/Cl co-transporter 2 (KCC2) in case of oxygen-glucose deficiency in cells. This phenomenon relies upon the strength of Zn^2+^ disclosure as temporary disclosure to high aggregation of Zn^2+^ cause signs of death, caspase determination in necrobiosis, while longer revelation to low aggregation of Zn^2+^ activates apoptotic, caspase-reliant cascades.

The caspase-mediated cell death happens through intrinsic way, which might be a result of extracellular-signal-regulated enzyme (ERK) inhibition responsible for caspase 3 activation and can cause down-directive of neuronal issues reliant opposed apoptotic genes (54). Several studies showed that Zn acts as a neuro-modulator, in contrast, a lot of experimental proofs have showed that the endogenous Zn might be comparatively forceful, speedily acting toxin, and to a minor level, conjointly a gliotoxin (55).

Zn binding and discharge from vesicles in presynaptic ends of a selected set of neurons that conjointly liberate salt. Consequently, these neurons are outlined as “gluzinergic” neurons (56). Zn will be discharged from presynaptic ends throughout conjugation transmission and enable to penetrate in postsynaptic stomata through specified gated channels holding N-methyl-D-aspartate (NMDA) (57). Zn exposure for 15 min at 300–600 μM to the nerve cells ends in animal neural death. It was assumed that a large amount of zinc was stored at the ends of neurons and that zinc sets free on depolarization a key role played by zinc in neuronal damage (58). Membrane depolarization is related to acute brain injury and it enhances the reactivity and efficiency of Zn to play role in neurolysin. Zn can be represented as a vital element of excitotoxic flow during trauma (56). Neuro-defense against Zn toxicity can be managed through the Zn chelators in the body cavity (59).

In addition to the effect of metallic elements on programmed cell death mentioned, zinc also induced programmed cell death in neurons that could be supported by two extra pathways. First, zinc-showing neurons association with receptor *p75*NTR and the *p75*NTR comprises of death executor the “NADE” as a mixture that can cause the cell death (60). Second, high intracellular metallic element deliberation activates the mitochondria leading to the discharge of pro-apoptotic proteins (61). Although the discharge of intracellular zinc activates neuronal cell death (62), signs of mortification, such as cell body puffiness and devastation of body organelles, have conjointly been discovered (63) showing that zinc-induced neuronal necrobiosis would possibly comprehend each apoptotic and death mechanisms (64).

Changes of neurotic Zn equilibrium affect the survival and Zn chelators acts as an agent for treatment of the stoke (65). It appears possible that Zn is additionally concerned with neuron damage diseases, e.g., Zn physiological state can be vital to the onset and for the development of Alzheimer's malady (66). The presence/application of metal chelators, such as clioquinol, to revive traditional somatic cell Zn physiological state has resulted in hopeful findings *in vivo* (67).

### Mechanism of Zinc in Oxidative Stress

**Figure 2:**
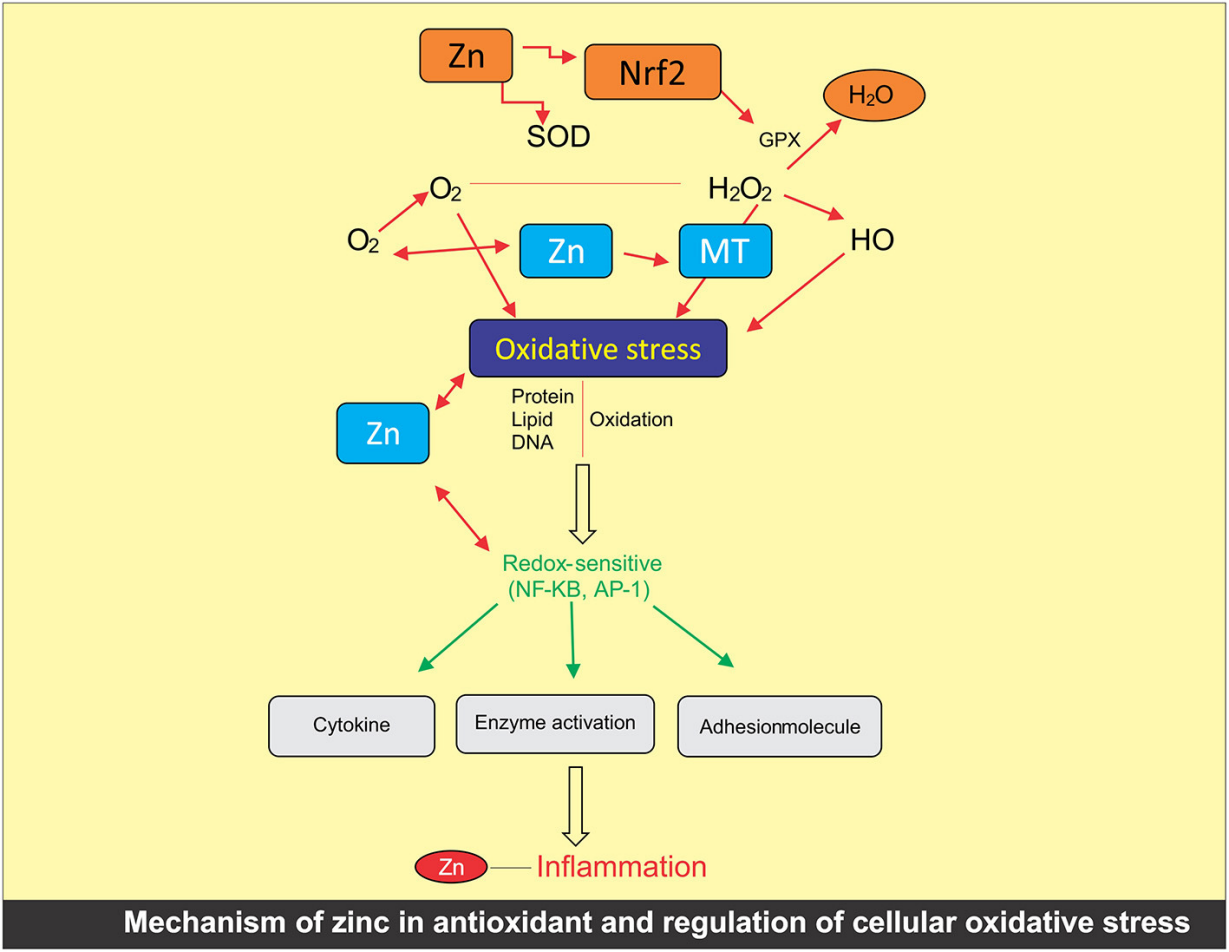
Mechanism of zinc in antioxidant and regulation of cellular oxidative stress.

The participation of Zn within the oxidative stress inhibitor has been widely studied (68). Investigations have revealed the importance of Zn as an antioxidant and co-factor for metallothionein proteins (Figure 2). However, in a semi-permeable membrane, the participation of Zn in combination with other essential metals, such as Fe and Cu, limits the NADPH oxidase catalyst and minimizes high sugar level, such as swellings (68). Zn as a structural component of the antioxidant enzyme superoxide dismutase (SOD) is a gift for cells to ameliorate oxidative damage through ROS. The presence of SOD in cell is responsible for converting two super oxide radicals into H_2_O_2_ and molecular oxygen, a possible ameliorating mechanism for ROS amelioration and cell protection (68). Because of this, regulating sufficient concentration of Zn within the cell is important for the proper antioxidant functioning. The experiments have shown that the deficiency of Zn may cause the distortion in SOD that persuades chronic endoplasmic reticulum pressure. As a result, this deficiency leads to impair protein manufacturing and induction of Zn carrier (69).

In another pathway, Zn acts as an inhibitor with the appearance of glutamate cysteine ligase. This causes the reduction of free radicals of Zn either directly with the help of glutathione or indirectly as a per-oxidase co-factor (70). Maintaining 100–150 mM of Zn within the refined human retinal stain somatic cells line adjusts the mRNA level through a related factor 2 (Nrf2)-dependent way. With this approach, Zn regulates the concentration of cellular glutathione (71).

Zinc is absolute to metallothionein below traditional physiological circumstances. In oxidative anxiety circumstances, the nutrient is discharged with metallothionein with decentralization in the cell. It also enhances the metallothionein expressions in the liver of rats for Zn supplementation, which inhibits the anti-inflammatory effects (72). The level of Zn controls the Zn^2+^ transporters and importer, which are the solute carrier family of 30A *ZnTs* and *39A ZIPs*, respectively (73). Additionally, Zn^2+^ is mobilized with the help of voltage-gated channels, glutamate, neurotransmitter receptors, and transporter proteins across the cellular membrane (74).

The higher Zn^2+^ levels within the cytoplasm are going to be known by the Zn^2+^ sensitive transcription issue, MTF-1, such as once Zn^2+^ translocated to the nucleus for upregulating the appearance of MT and ZnT (75). Otherwise, Zn^2+^ will be buffered at intervals a “Zn muffler” by unknown Zn^2+^ transporter and translocated to store the Zn in the mitochondria, endoplasmic reticulum, dictyosome, and lysosomes (76).

#### Role of Zinc as Either an Antioxidant With Regulation of Intracellular Free Zinc

Adverse effects of ROS can be prohibited or reduced by enhancing antioxidants activities in the cell, such as Mt, glutathione, ascorbate, and tocopherol (77). However, cellular Zn has the ability to reduce inflated levels of ROS through an antioxidant defense system. The extreme oxidation/peroxidation mainly happens *in vivo* in zinc-lacking tissues of animals (78). Dietary Zn shortage does not damage the general inhibitor protection capabilities in any tissue (79). The free fundamental defense system remains equipped with the antioxidants, which can be boosted by Zn supplementation (78).

The achievable pathway of ROS can be linked with six different processes: (i) the Cu/Zn-specific SOD inhibitor enzyme activity. There is no relationship between Cu/Zn-SOD with dietary intake of Zn concentration. SOD is not involved in the aerobic anxiety levels that is why its deficiency is not an important issue for aerobic stress. The modification of copper and Zn SOD is not happening with the help of Zn^2+^ in nutritional trauma conditions but somehow joined during Cu concentration. In contrast, mismetallothionein within the absence of Zn^2+^ could also be injurious yield with inactive macromolecule at the risk of accumulation; (ii) introduction of MT with the help of Zn^2+^ shows disturbances; (iii) free sulfhydryl group protection is lower in groups as Zn^2+^ will shield the sulfhydryl groups within the proteins against reaction. This has shown in dihydroorotase, Zn binding protein with finger DNA, and δ-amino levulinate dehydratase. As Zn^2+^ are lost from Zn-MT during the reaction with OH^−^ and ${\text{O}}_{2}^{-}$ that is why Zn does not shield all sulfhydryl groups against aerobic damage (78); (iv) less struggle with metal ions of redox-active during the aerobic reaction. Zn^2+^ contend with copper or iron on binding sites, attributable to the similarities in their management chemistry (80). Moreover, the efficiency of Zn^2+^ with copper and iron within the cell wall ends up in a reserve of the NADPH enzyme protein, another supply of O_2_ and H_2_O_2_ assembly and attenuates chronic irritation and symptoms (81); (v) destruction of the mitochondria or ER attributable under Zn deficiency (82); and (vi) indirect Zn involvement in aerobic reactions. The ionic zinc (Zn^2+^) binds by selection to NADPH instead of NADH. Therefore, Zn^2+^ will reduce the mediated drugs of NADPH.

## Approaches To Overcome the Deficiency of Zinc In Human Body

Shortage of Zn in humans can be compensated by adopting two possible strategies: (i) nutraceutical approach with the help of nutritional supplements and use of specific nutrients rich foods. (ii) Biofortification means enhancing Zn concentrations in food grains, achieved through propagation methods or by fertilizing crops with Zn supplementation.

### Nutraceutical Approach

Nutraceuticals are the chemicals other than human nutrition, which also can be used as medicine. Moreover, it does not only provide physiological benefits but also protects from Zn deficiency-related chronic illness. Supplementation basically refers to some highly absorbable pharmaceuticals, nutraceuticals, and chemical compounds of specific nature as capsules, syrups, and pills (83). Zinc supplementation to the infants is beneficial for the linear growth and development of infants along with treatment of child diarrhea (84). Supplementation in therapeutic and preventive forms is beneficial for the treatment of various infections and most commonly diarrhea. Preventive Zn supplementation reduces the occurrence of diarrhea up to 27% among children of age up to 12 months and reduces the occurrence of respiratory tract infections and reduces infants mortality up to 6% (84). In another study, it was observed that zinc and iron are the crucial micronutrients required for the infants in order to limit or curtail physical growth, reduce morbidity, and enhance psychomotor development (85).

Zinc supplementation programs face many challenges, such as product availability, treatment compliance, coverage, endorsement, and training (86). Zn-sulfate tablets are the most common nutraceutical mode used in the supplementation drive because of their equal importance and acceptance by the child and mothers, inexpensiveness, and ease in transportation and handling. Moreover, the dietary supplementations of Zn along with other micro-nutrients is another mode used for curing gastro-intestinal inflammatory ailments (GIIA) (87). The heterogeneous reactions of human body, different tissues, and curing multiple diseases by Zn emphasize to study the universal Zn supplementation as an emerging strategy more specifically for acute diarrhea treatment and respiratory infections among young children (88). International Zn nutrition-consultative group suggested that Zn nutrition is essential for the optimal growth and development and also enhances the pregnancy outcomes.

On the other hand, there might be some adverse effects of these synthetic nutraceuticals depending upon their manufacturing material. The nutraceuticals contain mycotoxins, pesticides, and heavy metal residues while manufacturing the supplementation from coffee beans (89), legumes (90), herbal plants (91), edible vegetable oil (92), and green tea (93). The present research is therefore focused on the biological methods (biofortified food consumption) to overcome the Zn deficiency in humans.

### Zinc Biofortification for Food Security

Food fortification is a sustainable and long-term resolution because of its ease in controlling precise micronutrient deficiencies among the population, especially young ones. According to the WHO, nutritional objective outlined as “the provision of most (97.5%) people within the population teams at maximum threat of shortage with associate degree sufficient ingestion of definite micronutrients, while not inflicting a hazard of extreme intakes in these or different groups” (83), it was decided to develop micronutrient biofortified food crops through selective breeding programs especially in cereals, i.e., wheat, rice, maize, and pearl millet, to overcome the micronutrients deficiencies in populations and next generations (86).

These fortification methods have the ability to increase the bound trace elements concentrations in cereal grains by application through micronutrient-specific fertilizers to the soil preferably iodine, and Zn, or fertigation to the crop leaves. The biofortification through genetic and agronomic methods in pulses and cereals is important to enhance micronutrient contents (30). Agronomic biofortification is achieved by enhancing soil Zn phyto-availability with the help of Zn fertilizer, whereas the genetic biofortification is based on enhancing Zn uptake mechanisms in the plant from the soil and its translocation in edible plant parts. Bhatt et al. (94) described that in most agricultural soils, there are sufficient Zn concentrations to feed the crops for several years, but the problem is with its phyto-availability, which can be improved by agronomic and other methods (94).

The agronomic methods include the application of Zn fertilizer preferably ZnSO_4_ for attaining higher Zn levels in grains but the problem of its bioavailability to plant arise due to its fixation in soil (95). Scientists are using different approaches to fortify Zn in cereals, legumes, and other food crops, such as foliar application (96), fertigation (97), and biofortification, through microbes (98, 99). Among them the microbial biofortification is found beneficial in improving Zn concentrations in cereals and other food crops. Hussain et al. (100) described various sources of Zn and Zn solubilizing bacteria for its biofortification in maize and found 4-fold increase in Zn concentration as compared to the uninoculated control treatments. Whereas, 70% of higher fortification in the form of Zn concentration was found in wheat through plant growth promoting rhizobacteria (PGPR) application along with Zn insoluble sources (101). Both cereals are the staple diet of the layman in developing and developed world and their fortification with essential micronutrients will help to overcome malnutrition without the costly nutraceuticals and their side effects.

## Conclusion and Future Prospects

The usefulness of Zn for all age groups with special emphasis on young ones and pregnant women in basal digestion, protection against pathogens, and nutrition-related diseases advocated its essentiality in daily meal. Moreover, Zn signaling in the different physiological processes and its role co-factor of enzymes in metabolic pathways further enhance its importance in human nutrition for the proper functioning of the body tissues and metabolism. Zinc is also important to play role in pathophysiology of certain diseases, such as cancer, obesity, and diabetes, the deficiency of Zn enhances the oxidative damage to the body leading toward cellular damage to chronic diseases.

Certain approaches do exist to overcome deficiencies and promote the micronutrients bioavailability in plant-based staples just as diets are utilized in developed nations. The ever-increasing population is at threat because of Zn deficiency worldwide but the situation is worse in the undeveloped and developing world. Bio fortification and zinc fortification are the best and most advanced methods to fulfill the nutritional needs of the burgeoning population. Therefore, future research will be directed toward the nutritional deficiencies especially related to micro-nutrients, Zn role in body defiance against oxidative damage at the molecular level, effective supplementation of Zn to cure acute and chronic diseases, genetic approaches need to emphasize not only on cereals but also on vegetables and fruits, and development of the effective Zn fertilizers to enhance zinc uptake efficiency in crop.

## Author Contributions

AH, SS, WJ, and XW contributed to the conception and formatting of the contents. MA, AD, NS, and AM organized the database. AH and SS wrote the first draft of the manuscript. SM, MI, MA, and AD wrote sections of the manuscript. All authors contributed to manuscript revision, read, and approved the submitted version.

## Conflict of Interest

The authors declare that the research was conducted in the absence of any commercial or financial relationships that could be construed as a potential conflict of interest.

## Publisher's Note

All claims expressed in this article are solely those of the authors and do not necessarily represent those of their affiliated organizations, or those of the publisher, the editors and the reviewers. Any product that may be evaluated in this article, or claim that may be made by its manufacturer, is not guaranteed or endorsed by the publisher.
